# Health Care Workers' Mental Health During the First Weeks of the SARS-CoV-2 Pandemic in Switzerland—A Cross-Sectional Study

**DOI:** 10.3389/fpsyt.2021.594340

**Published:** 2021-03-18

**Authors:** Sonja Weilenmann, Jutta Ernst, Heidi Petry, Monique C. Pfaltz, Onur Sazpinar, Samuel Gehrke, Francesca Paolercio, Roland von Känel, Tobias R. Spiller

**Affiliations:** ^1^Department of Consultation-Liaison Psychiatry and Psychosomatic Medicine, University Hospital Zurich, University of Zurich, Zurich, Switzerland; ^2^Center for Clinical Nursing Science, University Hospital Zurich, University of Zurich, Zurich, Switzerland; ^3^Department of Internal Medicine, University Hospital Zurich, University of Zurich, Zurich, Switzerland; ^4^Department of Psychology, University of Zurich, Zurich, Switzerland

**Keywords:** COVID-19, mental health, health care worker, burnout, pandemic, Switzerland

## Abstract

**Objective:** The current SARS-CoV-2 pandemic poses various challenges for health care workers (HCWs). This may affect their mental health, which is crucial to maintain high quality medical care during a pandemic. Existing evidence suggests that HCWs, especially women, nurses, frontline staff, and those exposed to COVID-19 patients, are at risk for anxiety and depression. However, a comprehensive overview of risk and protective factors considering their mutual influence is lacking. Therefore, this study aimed at exploring HCWs' mental health during the SARS-CoV-2 pandemic in Switzerland, investigating the independent effect of various demographic, work- and COVID-related factors on HCWs' mental health.

**Methods:** In an exploratory, cross-sectional, nation-wide online survey, we assessed demographics, work characteristics, COVID-19 exposure, and anxiety, depression, and burnout in 1,406 HCWs during the beginning of the SARS-CoV-2 pandemic in Switzerland. Network analysis was used to investigate the associations among the included variables.

**Results:** Women (compared to men), nurses (compared to physicians), frontline staff (compared to non-frontline workers), and HCWs exposed to COVID-19 patients (compared to non-exposed) reported more symptoms than their peers. However, these effects were all small. Perceived support by the employer independently predicted anxiety and burnout after adjustment for other risk factors.

**Conclusion:** Our finding that some HCWs had elevated levels of anxiety, depression, and burnout underscores the importance to systematically monitor HCWs' mental health during this ongoing pandemic. Because perceived support and mental health impairments were negatively related, we encourage the implementation of supportive measures for HCWs' well-being during this crisis.

## Introduction

Since December 2019, the world has witnessed a pandemic spread of SARS-CoV-2 with increasing numbers of patients suffering from COVID-19 (1, 2). This global public health crisis poses various challenges for health care workers (HCW) all around the world. During the first weeks of the pandemic, some HCWs worked additional hours to care for the high number of COVID-19 patients and put themselves at risk for infection, while others have seen their workload diminish due to public health-related measures enforced by authorities (3).

From research in HCWs, it is well-known that work-related stressors such as working overtime are associated with impaired mental health, for example, in the form of burnout, anxiety, and depression (4–7). Importantly, the consequences of reduced mental health not only affect HCWs themselves, but also their professional functioning including the quality of care they provide (5, 8–10). This is highly problematic, given that medical performance is essential to manage the consequences of this public health crisis.

The authors of a recent meta-analysis reported higher levels of psychological distress in HCWs working with infected patients during emerging virus outbreaks (11). Moreover, they identified several risk factors for psychological distress in HCWs, including working as a nurse and lack of organizational support. On the other hand, access to personal protective equipment and adequate time off work were found to act protectively (11). Regarding the current SARS-CoV-2 pandemic, an increasing number of studies reported on HCWs' mental health [e.g., (12, 13)]. A meta-analysis of this literature conducted in April 2020 reported a pooled prevalence of 23.2% for anxiety and 22.8% for depression (14). In accordance with Kisely and colleagues (11), subgroup analyses revealed a higher prevalence of symptoms of anxiety and depression among females and nurses.

However, one general limitation was that the majority of these studies were conducted in a single country, namely China (14). Furthermore, the commonly conducted, unadjusted subgroup comparisons (e.g., the prevalence of distress among nursing staff compared to physician) were prone to bias due to a high intercorrelation among the included risk factors (“working as a nurse” is likely confounded by the gender imbalance among nurses). Additionally, a comprehensive investigation of the interactions between risk and protective factors and their adjusted effect on mental health among HCWs during an emerging virus outbreak is lacking.

In this cross-sectional, nation-wide study, we assessed the mental health of physicians and nurses during the SARS-CoV-2 pandemic in Switzerland. In addition, we collected data on known risk and protective factors, including demographics (e.g., gender, profession, professional experience), work characteristics (e.g., availability of support, work hours), and COVID-19 exposure at work (e.g., exposure to COVID-19 patients, working as frontline staff). The data was collected between March 28 and April 4, 2020. At that time, Switzerland had among the highest per capita rate of COVID-19 cases in the world.

This exploratory study had three aims. First, we assessed HCWs' mental health by their levels of anxiety, depression, and burnout. Second, we aimed to compare the levels of symptoms between commonly investigated subgroups (e.g., frontline and non-frontline workers) in a pairwise manner to ensure comparability of our results with the previous literature. Third, we conducted a network analysis to provide a comprehensive overview of the adjusted effects of the various factors outlined above on HCWs' mental health.

## Method

### Procedure

This study had an explorative, cross-sectional design with a single period of data collection and was carried out as a fully anonymous online survey in German, French, and Italian. The survey was accessible through a link and could be filled out using a computer, tablet, or smartphone. Data from participants was saved and accessible for analysis only after full completion of the survey. However, some items (e.g., years of professional experience) were assessed using a text-field, which led to minor data loss due to wrong input from participants. For the group differences analyses, missing data was handled by pairwise deletion. For the network analysis, all participants with missing data were removed.

The ethics committee of the canton Zurich assessed the study and officially declared that the study did not fall within the scope of the Human Research Act (BASEC-Nr. Req-2020-00471). Therefore, no authorization from the ethics committee was required. Still, all participants were asked for their informed consent at the beginning of the survey. Data was collected between March 28 and April 4, 2020, starting 2 weeks after the federal council (constituting the collective head of state) categorized the situation as “extraordinary” (March 16, 2020) (15) and singed an executive order resulting in a partial “lockdown” (15).

### Participants

Inclusion criteria were (a) actively working as a nurse or physician in Switzerland and (b) being at least 18 years old. Participants older than 69 years old, the age of the latest official retirement in Switzerland, were excluded from the current analysis. We implement the recruitment as a non-targeted, snowball approach using mailing lists of hospitals and professional societies, social media, and personal contacts of the study team members, with a focus on reaching health care workers in all parts of Switzerland.

### Sample

We received a total of 1,533 completed questionnaires. Of these, 124 (8.1%) participants did not meet the inclusion criteria. Of the remaining 1,409 participants, 3 (0.2%) indicated their gender as “other” and were excluded from further analysis to ensure comparability of groups. This resulted in a final sample size of 1,406, of which 857 (61.0%) were physicians and 549 (39.0%) were nurses.

### Measurements

#### Demographics

Demographics included age (in years), gender (woman, man, and other), profession (physician, nurse, and other), professional experience (in years), and canton (corresponding to a federal state) in which participants worked.

#### Work Characteristics

Participants reported their average weekly work hours prior to the pandemic, their total work hours during the past seven days, and their average hours of sleep per night during the past 7 days. Furthermore, using several single item questions with a Likert scale from 1 = “not at all” to 7 = “absolutely,” participants rated the extent to which they generally felt well-equipped (e.g., with protective masks), well-supported by the authorities and employers, and well-informed (e.g., about the development of the pandemic) by the authorities and employers.

#### COVID-19 Exposure and Frontline/Non-frontline

Exposure to COVID-19 was assessed by several nominal questions (yes/no). First, participants indicated if they had experienced COVID-19 symptoms (e.g., fever, cough) since the beginning of the pandemic or if they had been tested positively for SARS-CoV-2. Second, they reported whether they had been exposed to suspected or confirmed COVID-19 patients during work, and third, whether they had been working in a clinical unit designated to the diagnosis and treatment of COVID-19 patients. Participants answering to the latter question affirmatively were considered as frontline workers, the others as non-frontline workers.

#### Mental Health

The *General Anxiety Disorder-7* [GAD-7 (16)], a 7-item questionnaire, was used to measure symptoms of anxiety. Symptoms of depression were measured with the 9-item *Patient Health Questionnaire-9* [PHQ-9 (17)]. Both questionnaires are validated and frequently used instruments to assess the self-reported symptom severity of generalized anxiety and depression (18, 19). In both questionnaires, individual symptoms are assessed by ratings on a 4-point Likert scale ranging from 0 = “not at all” to 3 = “nearly every day.” An overall score can be calculated by summing individual items. Consequently, the sum scores of the GAD-7 and PHQ-9 range from 0 to 21 and 0 to 27, respectively. Sum scores of 10 points or higher indicate clinically relevant symptoms, corresponding to a diagnosis of generalized anxiety disorder or a depressive episode (16–18). Burnout was assessed using a brief measurement tool for physician burnout developed and validated by West et al. (20). This tool consists of two single items derived from the *Maslach Burnout Inventory* [MBI (21, 22)] measuring emotional exhaustion and depersonalization, two cardinal dimensions of burnout. These items were rated on a 7-point Likert scale ranging from 0 = “never” to 6 = “daily” and summed to form a total score. The answer format of all questionnaires was adapted to measure symptoms within the past 7 days. The German, French, and Italian translations of the questionnaires provided by the corresponding manuals were used.

#### Statistical Analyses

Due to abnormally distributed data, continuous and ordinal items were described using the median and interquartile range [IQR; 25–75%] Categorical data were described with frequency (%). Accordingly, we used two-tailed Mann-Whitney-*U*-tests and chi-square-tests to assess differences between independent groups. The effect size of group differences of ordinal and continuous variables was assessed as rank biserial correlation. The significance level for all tests was set to alpha = 0.05. Given the explorative study design, *p-*values were not adjusted for multiple comparisons. Descriptive statistics and comparison of independent groups were conducted using JASP version 0.11 (23).

We performed a network analysis to assess the relationship of the variables used in the independent group comparisons (gender, profession, COVID-exposure, (non-)frontline workplace) with symptoms of anxiety, depression, and burnout controlling for their mutual effects and for the influence of additional known risk factors [work hours, professional experience, perceived support (5, 24)]. Due to the high intercorrelation among the items assessing perceived support, we only entered “perceived support from the employer” into the network analysis. This item was chosen because it can be considered an umbrella term that subsumes instrumental support (i.e., feeling well-equipped) and informational support (i.e., feeling well-informed) (25).

In the resulting network, variables are represented by nodes, and the edges between these variables represent relationships between the variables (in the case of continuous variables the edge is equal to the partial correlation between them) adjusted for the effect of all other variables included in the network. Prior to the network estimation, symptom overlap was tested using the default settings of the goldbricker function of the *networktools* package (26). No exclusion of symptoms was suggested. The network was estimated using a regularization technique based on the least absolute shrinkage and selection operator [LASSO (27, 28)], which sets very small edges to zero and thus reduces the false positive rate [for more details see Epskamp et al. (29)]. Stability and reliability analyses were conducted as recommended using the *bootnet* package (29). Network analysis was performed in the R statistical environment using the *mgm* (30) *qgraph* (31) and *bootnet* package (28).

## Results

### Overall Sample

Table 1 summarizes demographics, work characteristics, and COVID-19 exposure of the whole sample. Symptom severity scores are presented in Table 2. Of the finally included 1,406 participants, the majority were German-speaking (*n* = 1,120, 79.7%), women (*n* = 930, 66.1%), had a median age of 34 years [29–46] and a median professional experience of 10 years [4–20]. Median working hours in the sample was 45 [36–54], with 572 (40.7%) participants working more hours than before the pandemic. Overall, experienced availability of medical equipment, support, and information by the employer and the authorities was high (all median scores ranging between 5 and 6, with 7 indicating the upper bound of the scale). One hundred ninety-six (13.9%) of the participants had suspected COVID-19 symptoms or were tested positive for SARS-CoV-2, 1,101 (78.3%) had contact with COVID-19 patients at work and 654 (46.5%) worked in designated COVID-19 units. Median anxiety and depressive symptom scores were 6 [3–10] and 5 [2–9]. Hence, these median scores were in the mild range [5–9 points (16, 17)]. Based on the suggested cut-offs (a total score of ≥10), 364 participants (25.9%) had clinically relevant symptoms of anxiety and 290 (20.6%) had clinically relevant symptoms of depression (see Supplementary Table 1). For the 2-item burnout scale, scores ranged from 0 to 12, and the sample median was 4 [2–6].

**Table 1:** Demographics, work characteristics, and COVID-19 exposure of 1,406 health care workers.

	**Overall (*N* = 1,406)**	
**Variable**	**Median**	***IQR***
**Demographics**		
Age in years^a^	34	29–46
Women, n (%)	930	66.1
Professional experience in years^a^	10	4–20
Nurses, n (%)	549	39.0
Physicians, n (%)	857	61.0
German speaking, n (%)	1,120	79.7
French speaking, n (%)	143	10.1
Italian speaking, n (%)	143	10.1
**Work characteristics**		
Total working hours in the previous 7 days	45	36–54
Total working hours per week prior to the pandemic^b^	45	40–50
Working more during the pandemic than before, n (%)^b^	572	40.7
Working less during the pandemic than before, n (%)^b^	414	29.4
Average number of sleep hours in the previous 7 days^c^	7	6–7.5
Having access to medical equipment	5	3–6
Perceived support by employer^d^	6	4–7
Perceived support by authorities	5	3–6
Perceived passage of information by employer^e^	6	4–7
Perceived passage of information by authorities	5	4–6
**COVID-19 Exposure**		
Had suspected COVID-19 symptoms or tested positive for SARS-CoV-2, n (%)	196	13.9
Was exposed to suspected or confirmed COVID-19 patients at work, n (%)	1,101	78.3
Worked in at clinical unit designated to diagnosis and treatment of patients with suspected or confirmed COVID-19, n (%)	654	46.5

a *N = 1,382*;

b *N = 1,349*;

c N = 1,332

d *N = 1,278*;

e *N = 1,286*.

**Table 2:** Mental health of 1,406 health care workers and comparison across different subgroups.

		**Gender**			**Profession**			**Workplace**			**Exposure to COVID-19 patients**		
	**Overall** **(*N* = 1,410)**	**Women** **(*n* = 934)**	**Men** **(n= 476)**	**Effect size**	**Nurses** **(*n* = 553)**	**Physicians** **(*n* = 857)**	**Effect size**	**Frontline** **(*n* = 655)**	**Secondline** **(*n* = 755)**	**Effect size**	**Yes** **(*n* = 1,103)**	**No** **(*n* = 307)**	**Effect size**
Anxiety	6 (3-10)	6 (4–10.75)	4 (3-8)^***^	−0.197	7 (4-10)	5 (3-9)^***^	−0.144	6 (3-11)	5 (3-9)^**^	−0.095	6 (3-10)	5 (3-8)^***^	−0.155
Depression	5 (2–8.75)	6 (3-9)	4 (2-7)^***^	−0.165	6 (3-9)	5 (2-8)^***^	−0.140	6 (3-10)	4 (2-8)^***^	−0.146	6 (3-9)	4 (1-7)^***^	−0.220
Burnout	4 (2-6)	4 (2-6)	4 (2-6)		4 (2-7)	4 (2-6)^*^	−0.077	5 (2-7)	3 (2-6)^***^	−0.198	4 (2-7)	3 (1-5)^***^	−0.250

*ES, Effect Size; Effect size is measured as a rank-biserial-correlation; Burnout, Overall burnout symptom score; Anxiety, Overall GAD-7 score; Depression, Overall PHQ-9 score*.

* *p < 0.05*,

** *p < 0.01*,

*** *p < 0.001*.

### Group Differences

Results from group comparisons of symptom severity are presented in Table 2. In summary, women had higher symptom levels of anxiety and depression than men, yet similar burnout symptoms. Nursing staff showed more symptoms of anxiety, depression, and burnout than physicians. HCWs exposed to COVID-19 patients had more symptoms than non-exposed HCWs, and frontline staff showed more symptoms than non-frontline staff. However, all group differences showed small effects (ranging from −0.077 to −0.250). Similar to these results, a significantly higher share of women (than man), nurses (than physicians), frontline staff (than non-frontline staff), and HCWs exposed to COVID-19 patients (than the non-exposed to COVID-19 patients) had clinically relevant symptoms of anxiety and depression (see Supplementary Table 1).

### Relationships Among the Investigated Variables (Network Analysis)

**Figure 1:**
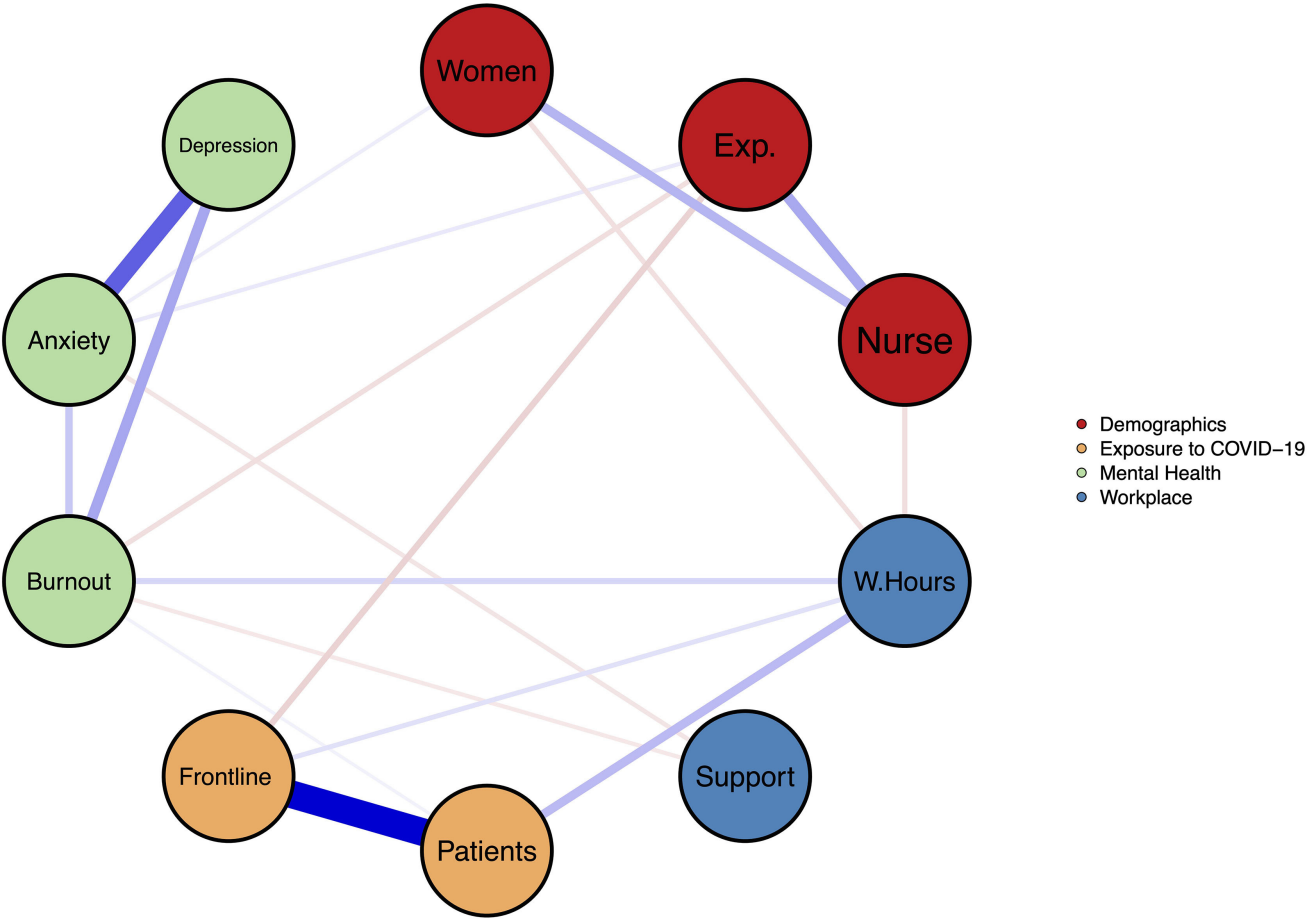
Relationships between demographics, work characteristics, COVID-19 exposure, and symptoms of anxiety, depression, and burnout. Nodes represent variables; Edges represent statistical associations between variables (blue = positive, red = negative, thickness = magnitude of the correlation); The coloring of the nodes indicates their class membership (Red = Demographic data, Blue = Workplace characteristics, Orange = Exposure to COVID-19, Green = Mental Health); Women = Gender (Levels: Men = 1, Women = 2); Exp. = Professional experience in years; Nurse = Nursing Staff (Variable = Profession. Levels: Physician = 1, Nurse = 2); W.Hours = Total working hours in the previous 7 days; Support = Perceived support by employer; Patients = Exposure to suspected or confirmed COVID-19 patients at work (Levels: No = 0, Yes = 1); Ward = Working in clinical unit designated to diagnosis and treatment of patients with suspected or confirmed COVID-19 (Levels: No = 0, Yes = 1); Burnout = Overall burnout symptom score; Anxiety = Overall GAD-7 score; Depression = Overall PHQ-9 score.

The results of the network analysis are visualized in Figure 1. The edges in the network represent statistical relationships between the variables, with the thickness of the edge representing the magnitude of the association and the color indicating the direction (red = negative, blue = positive). As expected, being a woman was associated with working as a nurse, and working in a designated COVID-19 unit was associated with exposure to COVID-19 patients at work. Moreover, the total symptom scores of anxiety, depression, and burnout were associated with one another. Symptoms of depression were not associated with any factor other than burnout and anxiety. Regarding anxiety, associations with gender, professional experience, and perceived support by the employer emerged. Burnout was associated with professional experience, work hours, exposure to COVID-19 patients, and perceived support from the employer. Additional results (e.g., stability analyses of the network) are presented in the Supplemental Digital Content.

## Discussion

In summary, participants reported mild levels of anxiety and depression, and elevated burnout scores. Approximately 40% of our sample worked more during the pandemic than before. Almost half of the sample was assigned to a designated COVID-19 unit and close to 80% were exposed to suspected or confirmed COVID-19 patients at work. Importantly, health care workers felt mostly well-equipped, supported, and informed by the employers and authorities.

At first glance, the prevalence of clinically significant anxiety and depression in our sample (25.9 and 20.6%) was comparable to the reported prevalence of anxiety (23.2%) and depression (22.8%) in the meta-analysis by Pappa et al. (14); (also see Supplementary Table 1). However, the inspection of the studies using the same questionnaires as we did, revealed a substantially higher the prevalence of anxiety (44.5%) in the study by Lai and colleagues (12). However, they defined relevant symptoms of anxiety as an overall score on the GAD-7 of seven or higher, whereas, we used the cut-off suggested by the developers of the GAD-7, namely ten (16). Applying the same cut-off of ten points to Lai et al.'s (12) study results in a prevalence of clinically relevant anxiety of 12.2% (154 of 1,257 participants), This is sustainably lower than in our sample. The higher prevalence of anxiety and depression among Swiss HCWs could have several reasons. First, at the time of data collection, Switzerland had a very high per capita rate of COVID-19 cases, higher than during the study period of the other two studies. Second, in non-pandemic times, several studies have consistently documented higher levels of anxiety and depression in European countries compared to Asian nations (32). Thus, the higher prevalence in our sample might be due to higher general levels of anxiety and depression or to culture-dependent social desirability effects. Third, in contrast to China, Switzerland was not affected by the SARS pandemic at the beginning of the century. Thus, Chinese HCWs probably had more experience dealing with a pandemic than Swiss HCWs, which could have reduced their symptom burden (33). These hypotheses are underscored by a study conducted among Italian HCWs during a time-period in which Italy had an even higher COVID-19 case-load per capita than Switzerland (34). The authors reported a median overall score of 9 [4–13] for the GAD-7 and of 10 [5–14] for the PHQ-9 among their participants, which was both above the corresponding medians in this study. Still, the generalizability of our findings to other (non-) European countries remains limited due to the different characteristics of the healthcare systems, governmental response to the pandemic and the course of the pandemic itself, which vary across different countries.

The group comparisons revealed that women (compared to men), nurses (compared to physicians), frontline staff (compared to non-frontline staff), and health care workers exposed to COVID-19 patients (compared to non-exposed), exhibited higher levels of symptoms and a higher prevalence of clinically relevant symptoms of anxiety and depression. However, all effects were small. The found differences between women and men, and nurses and physicians are in line with the subgroup analyses of the meta-analysis by Pappa et al. (14). However, as noted above, unadjusted comparisons across several groups must be interpreted with caution, because some variables (e.g., women and nurse or frontline workers and exposure to COVID-19 patients) are highly intercorrelated and thus confound results.

By conducting the first network analysis on mental health and associated factors during the pandemic, we were able to map the relationships between several variables while controlling for the mutual influence of all other variables included in the network. Here, we highlight four clusters of associations. First, being a frontline worker and exposure to COVID-19 patients was not associated with anxiety and depression when controlling for the effects of the other variables in the network. This relativizes the (unadjusted) effects found in the subgroup analyses, in which both, being a frontline worker and exposure to COVID-19 patients, was associated with more anxiety and depression. However, the relationship between exposure to COVID-19 patients and burnout was still significant. Second, support by the employer was a significant predictor of both burnout and anxiety. The role of perceived support has been studied extensively in occupational and health psychology (25), and its direct relationship with the mental health of HCWs is well-documented (5, 6, 35). For example, as postulated by the job-demand-control-support model (36), which has a broad empirical foundation (37), support is not only important to well-being but reduces the mental strain caused by job demands such as in this case, COVID-19 exposure. Moreover, the importance of support was also recently emphasized in a qualitative study on HCWs' concerns regarding the SARS-CoV-2 pandemic (38). Third, anxiety was associated with depression, burnout, female gender, longer professional experience, and perceived support from the employer. While higher levels of anxiety among women in general (39) and in female HCWs during a pandemic in particular (12, 40) are well-documented, the positive association with professional experience is counterintuitive. Given that HCWs with higher professional experience tended to be older, we can only speculate that these HCWs more likely belonged to a risk group for COVID-19 related complications (e.g., cardiac diseases). This might have led to more anxiety. Fourth, burnout was negatively associated with professional experience and positively related to work hours, which is well-documented in the literature on HCW burnout (5, 24).

This study is limited in several ways. First, the cross-sectional nature of the study with a single period of data collection and no control group does not allow to draw conclusions about the baseline prevalence of the investigated symptoms nor about their change. In other words, neither do we know whether symptoms changed compared to before the pandemic, nor whether HCWs reacted in a specific way differently from the general population. These important questions need to be addressed in future studies with an appropriate design (e.g., within the frame of ongoing cohort studies also including non-HCWs as a control group). Nevertheless, the symptom level of anxiety, depression and burnout in our population was comparable to the prevalence among HCWs in Switzerland in studies conducted during non-pandemic times (41–43). However, these studies used different instruments to assess symptom levels than the present study. Thus, symptom levels are not directly comparable. Second, given the non-targeted recruitment, our sample was likely not representative of HCWs in Switzerland. Moreover, the non-targeted recruitment might have introduced a selection bias in several ways (e.g., very busy HCWs or those with a higher symptom burden might not have been willing to participate). In addition, due to the non-targeted recruitment, we were unable to calculate a response rate. Still, the 857 physicians participating in this study represent ~2.3% of all 37,882 licensed Swiss physicians in 2019 (44). Third, the mental health of participants was measured using self-report questionnaires. This might lead to an overestimation of symptoms (17). In addition, several variables (e.g., perceived support) were assessed using non-validated, single item questions. Future research should aim to incorporate validated tools whenever possible. Fourth, the adaptation of all questionnaires to cover symptom experience over the last 7 days has not been validated and limits comparability to studies undertaken with the original validated versions of the questionnaires [covering 2 weeks in case of the GAD-7 (16) and PHQ-9 (17) and a full year in case of the brief measurement tool for physician burnout developed and validated by West et al. (20)]. However, the strength of the restriction to the past 7 days lies in the capacity to measure symptoms during a highly dynamic time of crisis. Finally, questions regarding COVID-exposure or perceived support were developed for this specific study and were therefore not validated.

Notwithstanding these limitations, our study has clinical and scientific implications. The relatively high percentage of HCWs with clinically relevant symptoms of anxiety (25.9%) and depression (20.6%) underscores the importance to systematically monitor HCWs' mental health during this ongoing pandemic. Furthermore, supportive measures should be implemented. Such measures should address the key concerns of HCWs identified in previous research [e.g., sufficient access to personal protective equipment and access to child-care during increased work hours (38)] or stress-induced symptoms [e.g., stress reduction coaching based on skills from different psychotherapy modalities (45)]. Most importantly, however, HCWs themselves can best express their individual needs. Hence, we encourage managers and regulators to actively engage with the health care force and actively address their concerns. Due to the well-documented negative effect of impaired mental health on HCWs on their provided care (5, 8–10), these measures not only support HCWs themselves but also serve patients by ensuring the continuation of high-quality care, especially during a public health crisis.

Regarding future research, the most important question is how the mental health of HCWs will develop over the course of the pandemic. This should be addressed by longitudinal studies, ideally using a cohort design with several assessment time points during and after the SARS-CoV-2 pandemic. In addition, existing cohort studies with HCWs could conduct an additional assessment during the pandemic and would then be able to assess whether HCWs developed more symptoms during the pandemic compared to before. Moreover, it has to be noted that mental health problems during a pandemic may not be the direct result of the pandemic itself, but rather due to the containment efforts (e.g., social isolation) or the economic consequences (e.g., job loss) (46). To what extent HCWs are less affected by these consequences (e.g., due to their job security) compared with the general population during this pandemic also warrants further investigation.

In conclusion, in our sample overall symptom levels of anxiety and depression were mild, and burnout was elevated. Still, symptoms of anxiety and depression were significantly higher than in a similar study conducted in China (12) but lower than in an Italian study (34), both conducted during the beginning of the SARS-CoV-2 pandemic. In general, participants felt well-equipped and well-supported by their employer and the authorities. Women reported more symptoms than men, nurses more than physicians, frontline staff more than those not working on the frontline, and HCWs exposed to COVID-19 more than non-exposed peers. However, these effects were all small and most of them did not remain significant after controlling for other factors within the network analysis. Importantly, whereas, COVID-19 exposure was only partially associated with burnout, perceived support by the employer independently predicted anxiety and burnout. Given that the SARS-CoV-2 pandemic is ongoing and its future progress unpredictable, we encourage the implementation of monitoring systems for HCWs' mental health and measures to maintain their well-being during this crisis.

## Data Availability Statement

The raw data supporting the conclusions of this article will be made available by the authors, without undue reservation.

## Ethics Statement

Ethical review and approval was not required for the study on human participants in accordance with the local legislation and institutional requirements. Written informed consent for participation was not required for this study in accordance with the national legislation and the institutional requirements.

## Author Contributions

SW and TS were responsible for initiating this study and assembling the research team and drafted the manuscript. MP, HP, and RK supervised the research project. SW, JE, RK, and TS were equally responsible for conceptualizing the study. SW, JE, OS, SG, and FP provided administrative and technical support. All authors were involved in data acquisition, its analysis and its interpretation. All authors revised the manuscript, assisted in the final conceptualization of the manuscript, and assisted in finalizing it for submission. All authors read and approved the final version of the manuscript and the Supplementary Material.

## Conflict of Interest

The authors declare that the research was conducted in the absence of any commercial or financial relationships that could be construed as a potential conflict of interest.

## Abbreviations

HCW: health care workers
SARS-CoV-2: Severe Acute Respiratory Syndrome Coronavirus 2
COVID-19: Coronavirus Disease 2019
GAD-7: General Anxiety Disorder-7
PHQ-9: Patient Health Questionnaire-9
MBI: Maslach Burnout Inventory
IQR: Interquartile range.

## References

[1] World Health Organization. Rolling Updates on Coronavirus Disease (COVID-19). (2020). Available online at: https://www.who.int/emergencies/diseases/novel-coronavirus-2019/events-as-they-happen (accessed February 01, 2021).

[2] World Health Organization. Coronavirus Disease (COVID-2019) Situation Reports. (2020). Available online at: https://www.who.int/emergencies/diseases/novel-coronavirus-2019/situation-reports/ (accessed February 01, 2021).

[3] von Matt R. Paradoxe Situation. Kurzarbeit in Spitälern in der Corona-Krise. (2020). Available online at: https://www.srf.ch/news/schweiz/paradoxe-situation-kurzarbeit-in-spitaelern-in-der-corona-krise (accessed February 01, 2021).

[4] Shanafelt TD Sloan JA Habermann TM. The well-being of physicians. Am J Med. (2003) 114:513–9. 10.1016/S0002-9343(03)00117-7 12727590

[5] Shanafelt TD Noseworthy JH. Executive leadership and physician well-being. Mayo Clin Proc. (2017) 92:129–46. 10.1016/j.mayocp.2016.10.004 27871627

[6] Wallace JE Lemaire JB Ghali WA. Physician wellness: a missing quality indicator. The Lancet. (2009) 374:1714–21. 10.1016/S0140-6736(09)61424-0 19914516

[7] Jennings BM. Work stress and burnout among nurses: role of the work environment and working conditions. In: Hughes RG, editor. Patient Safety and Quality: An Evidence-Based Handbook for Nurses Advances in Patient Safety. Rockville, MD: Agency for Healthcare Research and Quality. Available online at: http://www.ncbi.nlm.nih.gov/books/NBK2668/ (accessed April 26, 2020)

[8] Poghosyan L Clarke SP Finlayson M Aiken LH. Nurse burnout and quality of care: Cross-national investigation in six countries. Res Nurs Health. (2010) 33:288–98. 10.1002/nur.20383 20645421 PMC2908908

[9] Scheepers RA Boerebach BCM Arah OA Heineman MJ Lombarts KM. A Systematic review of the impact of physicians' occupational well-being on the quality of patient care. Int J Behav Med. (2015) 22:683–98. 10.1007/s12529-015-9473-3 25733349 PMC4642595

[10] Firth-Cozens J. Interventions to improve physicians' well-being and patient care. Soc Sci Med. (2001) 52:215–22. 10.1016/S0277-9536(00)00221-5 11144777

[11] Kisely S Warren N McMahon L Dalais C Henry I Siskind D. Occurrence, prevention, and management of the psychological effects of emerging virus outbreaks on healthcare workers: rapid review and meta-analysis. BMJ. (2020) 369:m1642. 10.1136/bmj.m1642 32371466 PMC7199468

[12] Lai J Ma S Wang Y Cai Z Hu J Wei N. Factors associated with mental health outcomes among health care workers exposed to coronavirus disease 2019. JAMA Netw Open. (2020) 3:e203976. 10.1001/jamanetworkopen.2020.3976 32202646 PMC7090843

[13] Tan BYQ Chew NWS Lee GKH Jing M Goh Y Yeo LLL. Psychological impact of the COVID-19 pandemic on health care workers in Singapore. Ann Intern Med. (2020) 173:317–20. 10.7326/M20-1083 32251513 PMC7143149

[14] Pappa S Ntella V Giannakas T Giannakoulis VG Papoutsi E Katsaounou P. Prevalence of depression, anxiety, and insomnia among healthcare workers during the COVID-19 pandemic: a systematic review and meta-analysis. Brain Behav Immun. (2020) 88:901–07. 10.2139/ssrn.3594632 32437915 PMC7206431

[15] Bundesamt für Gesundheit. Coronavirus: Bundesrat erklärt die ≪ausserordentliche Lage≫ und verschärft die Massnahmen. (2020). Available online at: https://www.bag.admin.ch/bag/de/home/das-bag/aktuell/medienmitteilungen.msg-id-78454.html (accessed February 01, 2021).

[16] Spitzer RL Kroenke K Williams JBW Löwe B. A brief measure for assessing generalized anxiety disorder: the GAD-7. Arch Intern Med. (2006) 166:1092. 10.1001/archinte.166.10.1092 16717171

[17] Kroenke K Spitzer RL Williams JBW. The PHQ-9: validity of a brief depression severity measure. J Gen Intern Med. (2001) 16:606–13. 10.1046/j.1525-1497.2001.016009606.x 11556941 PMC1495268

[18] Manea L Gilbody S McMillan D. Optimal cut-off score for diagnosing depression with the Patient Health Questionnaire (PHQ-9): a meta-analysis. Can Med Assoc J. (2012) 184:E191–6. 10.1503/cmaj.110829 22184363 PMC3281183

[19] Arroll B Goodyear-Smith F Crengle S Gunn J Kerse N Fishman T Falloon K. Validation of PHQ-2 and PHQ-9 to screen for major depression in the primary care population. Ann Fam Med. (2010) 8:348–53. 10.1370/afm.1139 20644190 PMC2906530

[20] West CP Dyrbye LN Sloan JA Shanafelt TD. single item measures of emotional exhaustion and depersonalization are useful for assessing burnout in medical professionals. J Gen Intern Med. (2009) 24:1318–21. 10.1007/s11606-009-1129-z 19802645 PMC2787943

[21] Maslach C Jackson SE. The measurement of experienced burnout. J Organ Behav. (1981) 2:99–113. 10.1002/job.4030020205

[22] Maslach C Jackson SE Leiter MP. Maslach Burnout Inventory Manual. 4th ed. Menlo Park, CA: Mind Garden (2018).

[23] JASP Team. JASP [Computer software]. (2020). Available online at: https://jasp-stats.org/ (accessed February 01, 2021).

[24] Stimpfel AW Sloane DM Aiken LH. The longer the shifts for hospital nurses, the higher the levels of burnout and patient dissatisfaction. Health Aff (Millwood). (2012) 31:2501–9. 10.1377/hlthaff.2011.1377 23129681 PMC3608421

[25] Taylor SE. Social support: a review. In: Friedman HS, editor. The Oxford Handbook of Health Psychology. Oxford: Oxford University Press.

[26] Jones P. Networktools: Tools for Identifying Important Nodes in Networks. (2019). Available online at: https://CRAN.R-project.org/package=networktools (accessed January 30, 2020)

[27] Epskamp S Waldorp LJ Mõttus R Borsboom D. The gaussian graphical model in cross-sectional and time-series data. Multivar Behav Res. (2018) 53:453–80. 10.1080/00273171.2018.1454823 29658809

[28] Epskamp S Fried EI. A tutorial on regularized partial correlation networks. Psychol Methods. (2018) 23:617–34. 10.1037/met0000167 29595293

[29] Epskamp S Borsboom D Fried EI. Estimating psychological networks and their accuracy: a tutorial paper. Behav Res Methods. (2018) 50:195–212. 10.3758/s13428-017-0862-1 28342071 PMC5809547

[30] Haslbeck JMB Waldorp LJ. mgm: Estimating Time-Varying Mixed Graphical Models in High-Dimensional Data. ArXiv151006871 Stat. (2020). Available online at: http://arxiv.org/abs/1510.06871 (accessed April 26, 2020). 10.18637/jss.v093.i08

[31] Epskamp S Cramer AOJ Waldorp LJ Schmittmann VD Borsboom D. qgraph: network visualizations of relationships in psychometric data. J Stat Softw. (2012) 48:1–18. 10.18637/jss.v048.i04

[32] WHO World Mental Health Survey Consortium. Prevalence, severity, and unmet need for treatment of mental disorders in the world health organization world mental health surveys. JAMA. (2004) 291:2581. 10.1001/jama.291.21.2581 15173149

[33] Tan CC. SARS in Singapore-key lessons from an epidemic. Ann Acad Med Singap. (2006) 35:345. 16830002

[34] Rossi R Socci V Pacitti F Di Lorenzo G Di Marco A Siracusano A. Mental health outcomes among frontline and second-line health care workers during the coronavirus disease 2019 (COVID-19) pandemic in Italy. JAMA Netw Open. (2020) 3:e2010185. 10.1001/jamanetworkopen.2020.10185 32463467 PMC7256664

[35] Velando-Soriano A Ortega-Campos E Gómez-Urquiza JL Ramírez-Baena L De La Fuente EI Cañadas-De La Fuente GA. Impact of social support in preventing burnout syndrome in nurses: a systematic review. Jpn J Nurs Sci. (2020) 17:e12269. 10.1111/jjns.12269 31617309

[36] Johnson JV Hall EM. Job strain, work place social support, and cardiovascular disease: a cross-sectional study of a random sample of the Swedish working population. Am J Public Health. (1988) 78:1336–42. 10.2105/AJPH.78.10.1336 3421392 PMC1349434

[37] Häusser JA Mojzisch A Niesel M Schulz-Hardt S. Ten years on: A review of recent research on the Job Demand–Control (-Support) model and psychological well-being. Work Stress. (2010) 24:1–35. 10.1080/02678371003683747

[38] Shanafelt T Ripp J Trockel M. Understanding and addressing sources of anxiety among health care professionals during the COVID-19 pandemic. JAMA. (2020) 323:2133–4 10.1001/jama.2020.5893 32259193

[39] McLean CP Asnaani A Litz BT Hofmann SG. Gender differences in anxiety disorders: prevalence, course of illness, comorbidity and burden of illness. J Psychiatr Res. (2011) 45:1027–35. 10.1016/j.jpsychires.2011.03.006 21439576 PMC3135672

[40] Zhang W Wang K Yin L Zhao W Xue Q Peng M. Mental health and psychosocial problems of medical health workers during the COVID-19 epidemic in China. Psychother Psychosom. (2020) 89:242–50. 10.1159/000507639 32272480 PMC7206349

[41] Arigoni F Bovier P Sappino A. Trend of burnout among Swiss doctors. Swiss Med Wkly. (2010) 140:w13070. 10.4414/smw.2010.13070 20809437

[42] Hämmig O. Gesundheit von Beschäftigten in Gesundheits-berufen. Zürich: Institut für Epidemiologie, Biostatistik und Prävention der Universität Zürich (2018).

[43] Buddeberg-Fischer B Stamm M Buddeberg C Klaghofer R. Angst und Depression bei jungen Ärztinnen und Ärzten - Ergebnisse einer Schweizer Longitudinalstudie. Z Für Psychosom Med Psychother. (2009) 55:37–50. 10.13109/zptm.2009.55.1.37 19353511

[44] Hostettler S Kraft E. FMH-Ärztestatistik 2019 – hohe Abhängigkeit vom Ausland. Schweiz Ärzteztg. (2020) 101:450–5. 10.4414/saez.2020.18725

[45] Rosen B Preisman M Hunter J Maunder R. applying psychotherapeutic principles to bolster resilience among health care workers during the COVID-19 pandemic. Am J Psychother. (2020) 73:144–8. 10.1176/appi.psychotherapy.20200020 32985915

[46] Brown S Schuman DL. Suicide in the time of COVID-19: a perfect storm. J Rural Health. (2021) 37:211–4. 10.1111/jrh.12458 32362027 PMC7267332

[47] Weilenmann S Ernst J Petry H Sazpinar O Pfaltz MC Gehrke S. Health care workers mental health during the first weeks of the SARS-CoV-2 pandemic in Switzerland: a cross-sectional study. medRxiv. (2020). 10.3389/fpsyt.2021.594340 PMC8012487 33815162

