# Supplementary material for: Health Care Workers' Mental Health During the First Weeks of the SARS-CoV-2 Pandemic in Switzerland—A Cross-Sectional Study
Source: Front Psychiatry. 2021 Mar 18;12:594340. doi: 10.3389/fpsyt.2021.594340 (PMC8012487; doi:10.3389/fpsyt.2021.594340)
Supplement: Supplementary file 1 [file Data_Sheet_1.DOCX]

**Supplemental Data File**

**Health Care Workers’ Mental Health During the First Weeks of the SARS-CoV-2 Pandemic in Switzerland – A Cross-Sectional Study**

**Table S1**
 *Clinically Relevant Symptoms of Anxiety and Depression of 1406 Health Care Workers Across Different Subgroups*

|  |  | Gender | | | Profession | | | Workplace | | | Exposure to COVID-19 Patients | | |
| --- | --- | --- | --- | --- | --- | --- | --- | --- | --- | --- | --- | --- | --- |
|  | Overall (N =1406) | Females  (n= 930) | Males  (n= 476) | *P*-value | Nurses (n= 549) | Physicians (n= 857) | *P*-value | Frontline (n= 654) | Secondline (n= 752) | *P*-value | Yes (n= 1101) | No (n= 305) | *P*-value |
| Clinically relevant Anxiety, n (%) | 364 (25.9) | 272 (29.2) | 92 (19.3) | <.001 | 160 (29.1) | 204 (23.8) | .026 | 198 (30.3) | 166 (22.1) | <.001 | 307 (27.9) | 57 (18.7) | <.001 |
| Clinically relevant Depression, n (%) | 290 (20.6) | 213 (22.9) | 77 (16.2) | .003 | 136 (24.8) | 154 (18.0) | .002 | 167 (25.5) | 123 (16.4) | <.001 | 246 (22.3) | 44 (14.4) | .002 |

*Note.* Effect size is measured as a rank-biserial-correlation; Clinically relevant anxiety = overall GAD-7 score ≥ 10; Clinically relevant depression = overall PHQ-9 score ≥ 10.

* p < 0.05, ** p < 0.01, *** p < 0.001

**Figure S1**

Bootstrap edge weights difference test between non-zero estimated edge-weights in the network shown in Figure 1.

*Note.* Bootstrapped difference tests (α = 0.05) between edge-weights that were non-zero in the network; Black boxes indicate significant differences between two edges, non-significant differences are indicated by grey boxes; the colour of the boxes (ranging from red to blue) corresponds to the magnitude of the edge (negative to positive).

Gender (Levels: Men = 1, Women = 2); Exp. = Professional experience in years; Prof = Profession (Levels: Physician = 1, Nurse = 2); W.Hours = Total working hours in the previous 7 days; Support = Perceived support by employer; Exp.Pat. = Exposure to suspected or confirmed COVID-19 patients at work (Levels: No=0, Yes= 1); C.Sta = Working in clinical unit designated to diagnosis and treatment of patients with suspected or confirmed COVID-19 (Levels: No=0, Yes= 1)

**Figure S2**

Bootstrap 95% confidence intervals for estimated edge weights of the network shown in Figure 1.


Each edge is represented by a horizontal line; Edge weights are represented by the red line; The grey area indicated the 95% confidence intervals.

Gender (Levels: Men = 1, Women = 2); Exp. = Professional experience in years; Prof = Profession (Levels: Physician = 1, Nurse = 2); W.Hours = Total working hours in the previous 7 days; Support = Perceived support by employer; Exp.Pat. = Exposure to suspected or confirmed COVID-19 patients at work (Levels: No=0, Yes= 1); C.Sta = Working in clinical unit designated to diagnosis and treatment of patients with suspected or confirmed COVID-19 (Levels: No=0, Yes= 1).
